# A kinetic study of biotransformation from valencene to nootkatone in an oscillatory baffled reactor

**DOI:** 10.1002/jctb.7350

**Published:** 2023-03-08

**Authors:** Mohamed Chilmeran, Matthew Hodges, Xiong‐Wei Ni

**Affiliations:** ^1^ Centre of Oscillatory Baffled Reactor Advancement (COBRA), School of Engineering and Physical Sciences Heriot‐Watt University Edinburgh UK; ^2^ Oxford Biotrans Ltd Thame UK

**Keywords:** nootkatone, valencene, enzyme, oscillatory baffled reactor, kinetics, mass transfer, biotransformation

## Abstract

In this paper we report our kinetic study of an oxidation reaction from valencene to nootkatone using enzyme in an oscillatory baffled reactor. The aims of this work are to elucidate the reaction mechanism and evaluate reaction kinetics. Towards these objectives, a full kinetic model using the Langmuir–Hinshelwood method was established and applied to the experimental data, allowing reactor schemes and orders to be confirmed and reaction rate constants to be extracted. Our full kinetic analysis suggests that most of the reversible reaction steps can be treated as irreversible, simplifying the overall reaction schemes. The effect of mass transfer on the kinetics was also investigated. © 2023 The Authors. *Journal of Chemical Technology and Biotechnology* published by John Wiley & Sons Ltd on behalf of Society of Chemical Industry (SCI).

## INTRODUCTION

Nootkatone is a highly valuable chemical due to its unique smell and flavour of grapefruit, and has been used in the food, beverage and fragrance industries. Nootkatone also shows a high insect‐repellent activity against different types of bugs.^1^ Nootkatone is found in grapefruit in trace amount; to extract 1 kg of nootkatone requires about 400 000 kg of grapefruit, which is clearly not sustainable, given the fact that grapefruit production has been in decline over the last 15 years, while demand for the flavour remains high.^2^ This has driven intensive research and development in producing nootkatone from chemically^3^, ^4^, ^5^ to biochemically,^6^ in order for nootkatone produced to be marketed as a natural product. The P450 enzyme family is one of the biocatalysts and is synthesized in biotechnological processes involving bacteria, fungi or plants. Valencene, being an essential oil having a flavour and taste of orange, is the main reactant. By introducing an oxygen atom into the allyl group of the valencene structure, it is then biotransformed to nootkatone due to the similarity between the chemicals' structures. Because of the widespread production and availability of orange, valencene can readily be extracted from orange peels or leftovers,^7^ and production of nootkatone from valencene has become the favourable route in chemical synthesis, in spite of the long reaction times, low conversions and low selectivity.^2^, ^8^, ^9^ A bioprocess using enzyme to produce nootkatone is a major competitor to chemical synthesis, which is the focus of this work.

**Figure 1:**
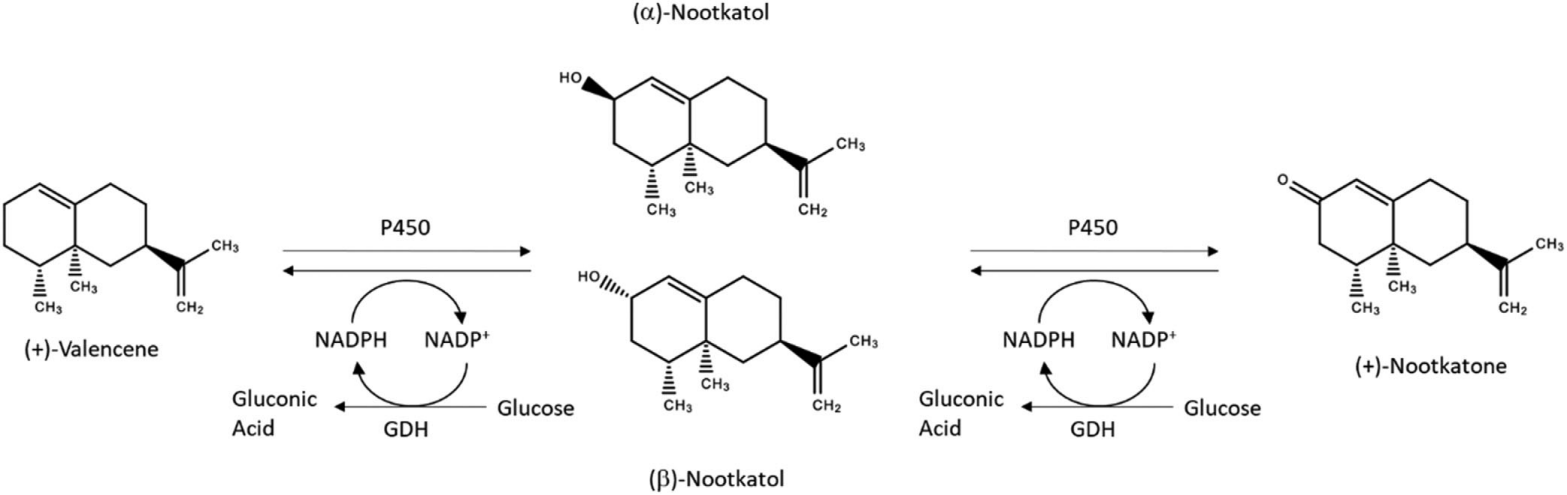
Reaction pathway of oxidation of valencene to nootkatone.

The reaction in question is an oxidation reaction where valencene is catalysed by a P450_BM3_ enzyme to form nootkatol, an alcohol in the form of two isomers, *α*‐nootkatol and *β*‐nootkatol. Further reaction between nootkatol and P450_BM3_ produces nootkatone, which is a ketone from *α*‐nootkatol. Small amounts of nootkatone epoxide are also produced as a by‐product in the final step of the reaction. Figure 1 shows the reaction pathway.

Different types of reactors have been used for the biotransformation of valencene to nootkatone in the past, including partitioning bioreactor, stirred tank reactor/bioreactor (STR/B), membrane aerated biofilm reactor and continuous flow microreactor.^10^, ^11^, ^12^, ^13^ Among these, the STR/B is one of the most used reactors due to the benefits of higher mass transport rate compared to the rest of the reactors.^14^ In the work reported here, an oscillatory baffled reactor (OBR) was employed, because the overall gas–liquid volumetric mass transfer coefficient in an OBR is six times higher than that in a bubble column and 75% higher than that of a stirred tank fermenter,^15^ due to more uniform and smaller bubble size, higher gas hold up and prolonged residence time of bubbles caused by oscillatory motions, leading to enhanced mass transfer characteristics.^16^, ^17^, ^18^, ^19^, ^20^, ^21^, ^22^, ^23^, ^24^ While there are publications concerned with the family of the reaction, few have involved kinetics. The focuses of the work reported here were to establish full reaction kinetics associated with the specific enzyme, and to evaluate the effect of mass transfer on the kinetics.

## EXPERIMENTAL AND ANALYTICAL METHODS

The following materials were used in the study.

*Enzyme*. The catalyst used in this work is a proprietary semi‐purified modified P450_BM3_ enzyme, produced through fermentation using an *E. coli* host and an associated supporting agent, both being supplied by Oxford Biotrans.

*Valencene*. Valencene is the main reactant and was purchased from De Monchy Aromatics with valencene purity by GC 70% area. It is an oily liquid of light orange colour with a smell similar to that of orange.

*Nictotinamide adenine dinucleotide phosphate (NADP+)*. NADP+ is a white powder used as a cofactor to donate hydrogen electrons in the reaction. The chemical was purchased from Glentham Scientific with ≥95% HPLC purity.

*Glucose dehydrogenase (GDH)*. This is a golden‐coloured powder used for regenerating NADP+ during oxidation and was purchased from CDX‐901 Glucose dehydrogenase901 (Codexis), optimized enzyme.

*Glucose monohydrate*. This white powder was used with GDH in the regeneration of NADP+ during oxidation and was purchased from Fisher Scientific.

*Buffers*. Buffers consist of monobasic potassium phosphate and dibasic potassium phosphate, both of which were purchased from Fisher Scientific. The former appears as small white crystals, and the latter as white powder.

Analytical materials include high‐performance liquid chromatography (HPLC)‐grade methanol for stopping reactions in samples and distilled water.

### Experimental setup

**Figure 2:**
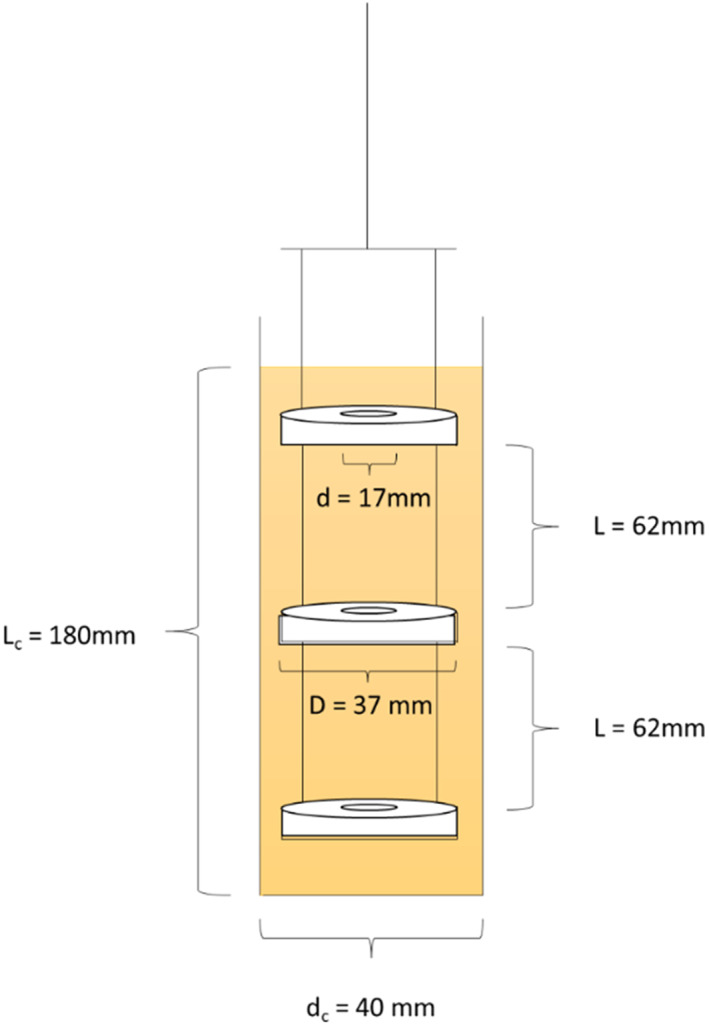
Setup of a batch OBR.

The OBR consisted of a cylindrical column of 40 mm in diameter (*d*_c_) and 210 mm long (*L*_c_), giving a volume of 300 mL and a working volume of 200 mL. The baffle string contained three orifice baffles with the following dimensions: baffle outer diameter (*D*) = 37 mm, baffle hole diameter (*d*) = 17.5 mm, baffle thickness (*t*) = 3 mm and baffle spacing (*L*) = 62 mm, as shown in Fig. 2. The baffle string was connected to a linear motor and drive, which provides the required oscillation frequency and amplitude.

### Procedure

An amount of 200 mL of purified water was mixed with enzyme‐associated support agents (glucose monohydrate, dibasic potassium phosphate and monobasic potassium phosphate). The pH was controlled between 7.6 and 7.8 at room temperature. The solution was then mixed with 3.3 mL of enzyme corresponding to an enzyme concentration of 1.5 μmol L^−1^. To avoid any potential loss of enzyme activity, valencene of a concentration of 6 mmol L^−1^ was added almost immediately. To start the reaction, NADP+ and GDH were finally added. The mass of NADP+ and GDH corresponds to 0.0347 and 0.024 wt% respectively. All reactions were done in an open reactor system.

### Characterization method

A HPLC instrument (Agilent 1100 Series) was used in the study. The column was a Zorbax Eclipse Plus C18 (4.6 × 100 mm, 3.5 μm) from Agilent Technologies. The mobile phase consisted of HPLC‐grade purified water with 0.05% acetic acid in line A and HPLC‐grade methanol in line B and flowed in at 1 mL min^−1^. An amount of 10 μL of sample was injected, while the column oven temperature was maintained at 40 °C and the wavelength of the ultraviolet detector was 210 nm.

**Figure 3:**
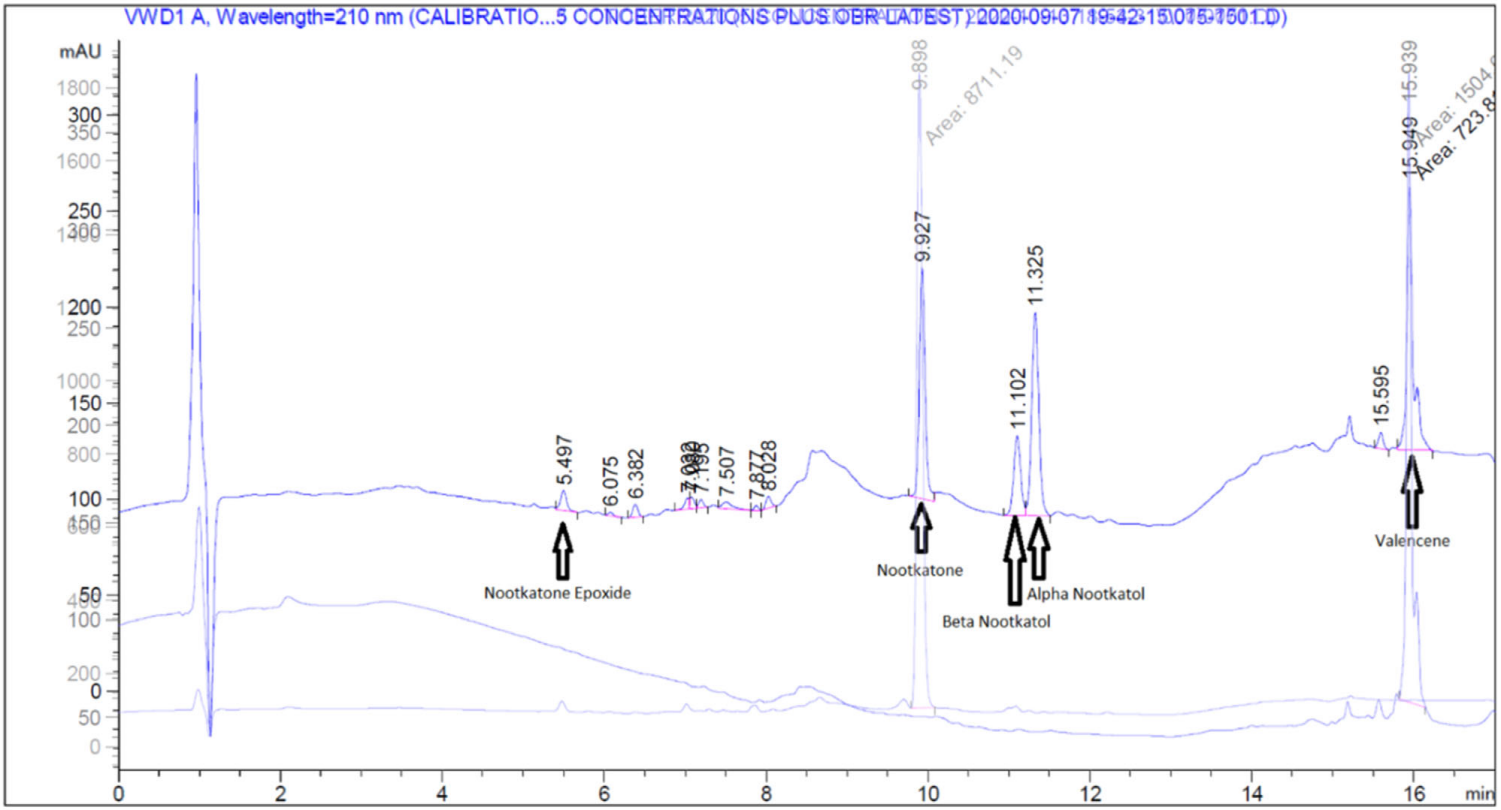
Three overlapping HPLC graphs of normal sample, pure valencene and pure nootkatone.

Two blank samples of either pure methanol or a mixture of 1:1 methanol and water were used to ensure that the system was properly equilibrated together with using pure methanol to clean the injection needle. The rest of the samples were added to the HPLC magazine after the blank samples. Figure 3 depicts a typical sample HPLC graph showing the peaks of each compound. Table 1 summarizes the peaks and approximate retention times where nootkatone is the main product; the derivatives of nootkatone are the intermediates.

**Table 1:** Peaks and retention times of components in HPLC

Peak	Approximate retention time (min)
Valencene	15.9
*α*‐Nootkatol	11.3
*β*‐Nootkatol	11.1
Nootkatone	9.9
Nootkatone epoxide	5.5

## RESULTS AND DISCUSSION

**Figure 4:**
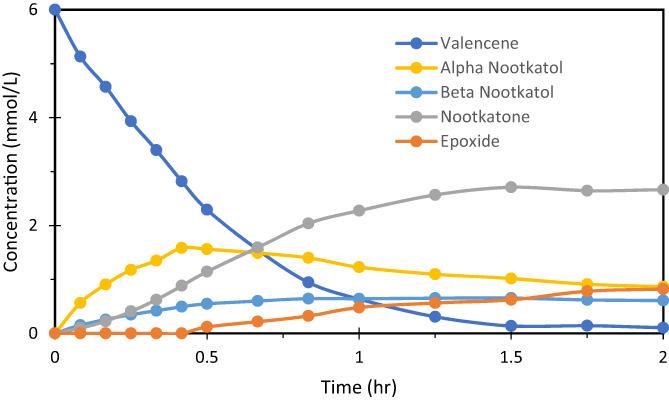
Concentration profile in the OBR (valencene concentration = 6 mmol L^−1^, enzyme concentration = 1.5 μmol L^−1^, aeration = 0 vvm, *f* = 2.5 Hz, *x*_o_ = 40 mm).

Figure 4 shows the concentration profiles in the OBR with valencene decreasing and nootkatone increasing with time, together with intermediates and by‐product. The bioconversion of nootkatone is about 40% in 1–1.5 h.

In order to carry out kinetic analysis, a kinetic model, based on the Langmuir–Hinshelwood kinetics formalism, was proposed, which accounts for one type of active enzyme site. The following assumptions were considered:

- Oxygen is supplied in excess (this is the case as surface air was entrained continuously).
- The system is operated isothermally.
- No evaporation of substrate or product occurs.
- The enzyme does not encounter any sheer damage due to oscillation.
- The aqueous phase and the organic phase (enzyme) were considered as pseudo‐homogeneous phase.

**Figure 5:**
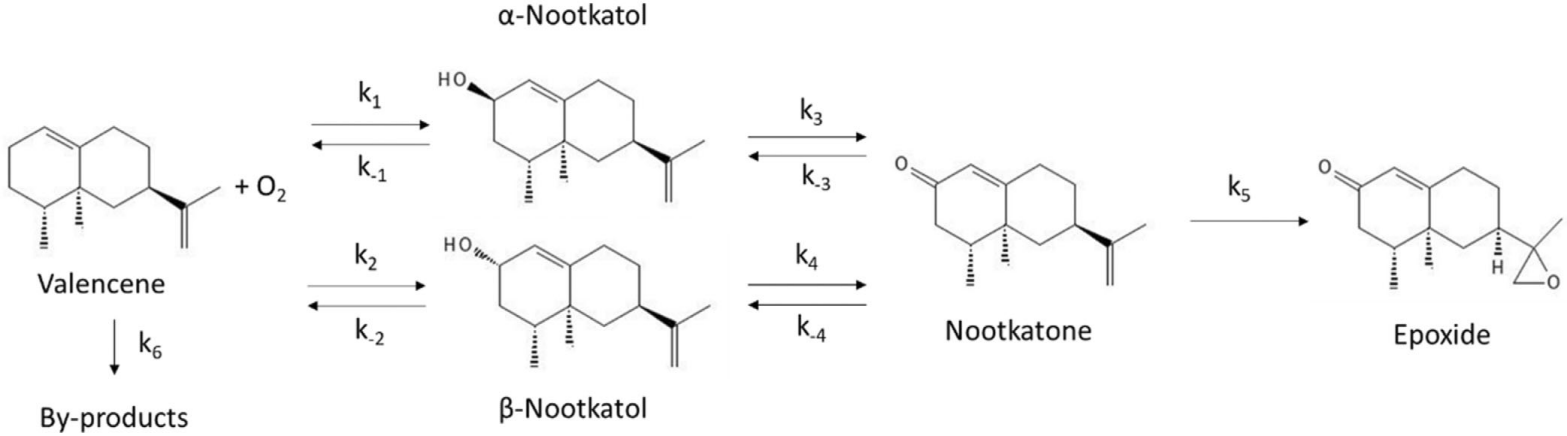
Overall reaction scheme.

The reaction scheme with reaction rate constants is shown in Fig. 5, consisting of four first‐order reversible reaction steps (*k*_1_/*k*_−1_, *k*_2_/*k*_−2_, *k*_3_/*k*_−3_, *k*_4_/*k*_−4_) and of two first‐order irreversible reaction steps (*k*_5_, *k*_6_). Similar reaction scheme is seen elsewhere.^2^, ^14^, ^25^ The kinetic expressions for each step are presented in Table 2, where *C* denotes the concentration of species, *θ* the mass balance of the surface coverage on the enzyme, *k* the rate constants, *K* the adsorption rate constants and *r* the rate of reaction step, and the subscripts V, A, B, N, E and By stand for valencene, *α*‐nootkatone, *β*‐nootkatone, nootkatone, epoxide and by‐product respectively.

**Table 2:** Elementary reaction steps for biotransformation of valencene to nootkatone

Valencene↔α−nootkatol	
$\begin{array}{c} V+•↔V• \end{array}$	$\begin{array}{c} K_{V}=\frac{\theta_{V}}{C_{V}\theta_{•}} \end{array}$
$\begin{array}{c} V•+O_{2}↔A• \end{array}$	$\begin{array}{c} r_{1}=k_{1}\theta_{V}-k_{-1}\theta_{A} \end{array}$
$\begin{array}{c} A•↔A+• \end{array}$	$\begin{array}{c} K_{A}=\frac{\theta_{A}}{C_{A}\theta_{•}} \end{array}$

Valencene↔β−nootkatol	
$\begin{array}{c} V+•↔V• \end{array}$	$\begin{array}{c} K_{V}=\frac{\theta_{V}}{C_{V}\theta_{•}} \end{array}$
$\begin{array}{c} V•+O_{2}↔B• \end{array}$	$\begin{array}{c} r_{2}=k_{2}\theta_{V}-k_{-2}\theta_{B} \end{array}$
$\begin{array}{c} B•↔B+• \end{array}$	$\begin{array}{c} K_{B}=\frac{\theta_{B}}{C_{B}\theta_{•}} \end{array}$

α−Nootkatol↔nootkatone	
$\begin{array}{c} A•↔N• \end{array}$	$\begin{array}{c} r_{3}=k_{3}\theta_{A}-k_{-3}\theta_{N} \end{array}$
$\begin{array}{c} N•↔N+• \end{array}$	$\begin{array}{c} K_{N}=\frac{\theta_{N}}{C_{N}\theta_{•}} \end{array}$

β−Nootkatol↔nootkatone	
$\begin{array}{c} B•↔N• \end{array}$	$\begin{array}{c} r_{4}=k_{4}\theta_{B}-k_{-4}\theta_{N} \end{array}$
$\begin{array}{c} N•↔N+• \end{array}$	$\begin{array}{c} K_{N}=\frac{\theta_{N}}{C_{N}\theta_{•}} \end{array}$

Nootkatone→epoxide	
$\begin{array}{c} N•\to E• \end{array}$	$\begin{array}{c} r_{5}=k_{5}\theta_{N} \end{array}$
$\begin{array}{c} E•↔E+• \end{array}$	$\begin{array}{c} K_{E}=\frac{\theta_{E}}{C_{E}\theta_{•}} \end{array}$

Valencene→by−products	
$\begin{array}{c} V•\to \mathrm{By}• \end{array}$	$\begin{array}{c} r_{6}=k_{6}\theta_{V} \end{array}$
$\begin{array}{c} \mathrm{By}•↔\mathrm{By}+• \end{array}$	$\begin{array}{c} K_{\mathrm{By}}=\frac{\theta_{\mathrm{By}}}{C_{\mathrm{By}}\theta_{•}} \end{array}$

The rate equations for each step are presented in Table 3. Mass transport limitations were considered in the model by the adsorption rate constants (*K*) together with the mass balance of surface coverage of the enzyme by the reaction species as expressed below:

(1) $$1=\theta_{•}+\theta_{V}+\theta_{A}+\theta_{B}+\theta_{N}+\theta_{E}+\theta_{\mathrm{By}}$$

**Table 3:** Reaction rate equations based on the Langmuir–Hinshelwood method

Reaction	Rate equation	
V↔A	$\begin{array}{c} r_{1}=\frac{k_{1}K_{V}C_{V}-k_{-1}K_{A}C_{A}}{1+K_{V}C_{V}+K_{A}C_{A}+K_{B}C_{B}+K_{N}C_{N}+K_{E}C_{E}+K_{\mathrm{By}}C_{\mathrm{By}}} \end{array}$	(3)
V↔B	$\begin{array}{c} r_{2}=\frac{k_{2}K_{V}C_{V}-k_{-2}K_{B}C_{B}}{1+K_{V}C_{V}+K_{A}C_{A}+K_{B}C_{B}+K_{N}C_{N}+K_{E}C_{E}+K_{\mathrm{By}}C_{\mathrm{By}}}\; \end{array}$	(4)
A↔N	$\begin{array}{c} r_{3}=\frac{k_{3}K_{A}C_{A}-k_{-3}K_{N}C_{N}}{1+K_{V}C_{V}+K_{A}C_{A}+K_{B}C_{B}+K_{N}C_{N}+K_{E}C_{E}+K_{\mathrm{By}}C_{\mathrm{By}}}\; \end{array}$	(5)
B↔N	$\begin{array}{c} r_{4}=\frac{k_{4}K_{B}C_{B}-k_{-4}K_{N}C_{N}}{1+K_{V}C_{V}+K_{A}C_{A}+K_{B}C_{B}+K_{N}C_{N}+K_{E}C_{E}+K_{\mathrm{By}}C_{\mathrm{By}}}\; \end{array}$	(6)
N→E	$\begin{array}{c} r_{5}=\frac{k_{5}K_{N}C_{N}}{1+K_{V}C_{V}+K_{A}C_{A}+K_{B}C_{B}+K_{N}C_{N}+K_{E}C_{E}+K_{\mathrm{By}}C_{\mathrm{By}}}\; \end{array}$	(7)
V→By	$\begin{array}{c} r_{6}=\frac{k_{6}K_{\mathrm{By}}C_{\mathrm{By}}}{1+K_{V}C_{V}+K_{A}C_{A}+K_{B}C_{B}+K_{N}C_{N}+K_{E}C_{E}+K_{\mathrm{By}}C_{\mathrm{By}}}\; \end{array}$	(8)

The coverage of active sites is expressed through the adsorption constants for the reversible steps as follows:

(2) $$\begin{array}{c} \theta_{•}=\frac{1}{1+K_{V}C_{V}+K_{A}C_{A}+K_{B}C_{B}+K_{N}C_{N}+K_{E}C_{E}+K_{\mathrm{By}}C_{\mathrm{By}}}\; \end{array}$$

The mole balances for the reactant and products are expressed as follows:

(9) $$\begin{array}{c} \frac{dC_{v}}{dt}=-r_{1}-r_{2}-r_{6} \end{array}$$

(10) $$\begin{array}{c} \frac{dC_{a}}{dt}=r_{1}-r_{3} \end{array}$$

(11) $$\begin{array}{c} \frac{dC_{b}}{dt}=r_{2}-r_{4} \end{array}$$

(12) $$\begin{array}{c} \frac{dC_{n}}{dt}=r_{3}+r_{4}-r_{5} \end{array}$$

(13) $$\begin{array}{c} \frac{dC_{e}}{dt}=r_{5} \end{array}$$

(14) $$\begin{array}{c} \frac{dC_{\mathrm{By}}}{dt}=r_{6} \end{array}$$

The objective function to assess the fitting of the kinetic model is shown below:

(15) $$\begin{array}{c} E\left(P\right)=\sum_{i,j}w_{j}∙{\left(x_{i,j}-y_{i,j}\left(P\right)\right)}^{2} \end{array}$$

where *P* is the tested parameter set (in this case the *k* values), *x*_*i*,*j*_ is a point in the dataset (experimental), *y*_*i*,*j*_(*P*) is the corresponding simulated value, *i* and *j* denote the rows and columns in the dataset and *w*_*j*_ is the weighting for each data column, and, for this test, the weighting is chosen to be the inverse of the mean square:

(16) $$\begin{array}{c} w_{j}=\frac{1}{<x_{j}^{2}>} \end{array}$$

**Figure 6:**
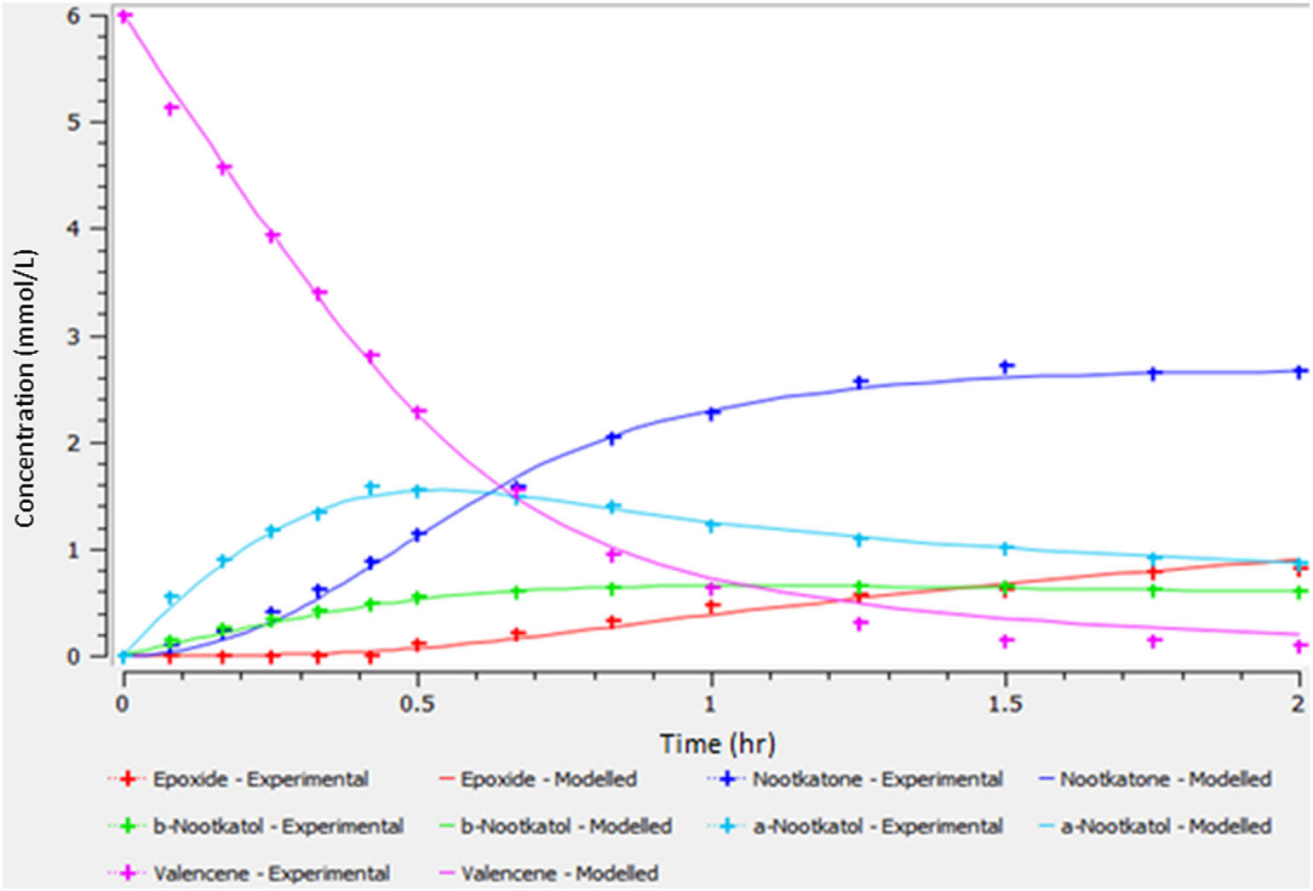
Fit between experimental data and model predictions (valencene concentration = 6 mmol L^−1^, enzyme concentration = 1.5 μmol L^−1^, aeration = 0 vvm, *f* = 2.5 Hz, *x*_o_ = 40 mm).

The aim of the objective function is to minimize the difference between experimental and simulated data. The system comprising of Eqns (3)–(14) was solved numerically using the parameter estimation function in Complex Pathway Simulator (COPASI) software. The initial concentrations of the species are given as *C*_v_ = 6 mmol L^−1^, *C*_a_ = *C*_b_ = *C*_n_ = *C*_e_ = *C*_By_ = 0 mmol L^−1^. Once a satisfactory level has been reached, e.g. objective value of 0.150, root mean square of 0.0417, error mean of 0.00116 and error mean standard deviation of 0.044, the series of *k* values are obtained. The fitting is shown in Fig. 6, where a good fit is seen for all species, confirming the reaction schemes and the orders of the reactions. The extracted rate constants are given in Table 4.

**Table 4:** Estimated kinetic parameters and adsorption constants (valencene concentration = 6 mmol L^−1^, enzyme concentration = 1.5 μmol L^−1^, aeration = 0 vvm, *f* = 2.5 Hz, *x*_o_ = 40 mm)

Model parameter	Value	Unit
*k*_1_	6.21	h^−1^
*k*_−1_	0.00	h^−1^
*k*_2_	1.37	h^−1^
*k*_−2_	0.76	h^−1^
*k*_3_	123.62	h^−1^
*k*_−3_	14.83	h^−1^
*k*_4_	6.46	h^−1^
*k*_−4_	0.00	h^−1^
*k*_5_	8.37	h^−1^
*k*_6_	1.17	h^−1^
*K*_A_	0.41	L mmol^−1^
*K*_B_	0.96	L mmol^−1^
*K*_E_	40.57	L mmol^−1^
*K*_N_	0.75	L mmol^−1^
*K*_V_	4.70	L mmol^−1^
*K*_By_	0.00	L mmol^−1^

**Figure 7:**
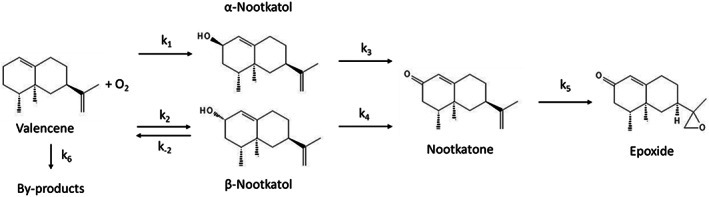
Simplified reaction scheme for biotransformation of valencene using P450.

From Table 4, we can see that three out of the four reversible reaction steps can be treated as irreversible, because *k*_1_ >> *k*_−1_, *k*_3_ >> *k*_−3_, *k*_4_ >> *k*_−4_, with *α*‐nootkatol to nootkatone (*k*_3_) as the dominant reaction with the largest *k* value. This would indicate that the P450 enzyme shows a preference more towards *α*‐nootkatol than *β*‐nootkatol (*k*_1_ > *k*_2_). For the irreversible steps, *k*_5_ (nootkatone to epoxide) is much higher than *k*_6_ (direct decomposition of valencene to by‐products), which is consistent with the concentration profiles shown in Fig. 4 where no by‐product was detected. The reaction of valencene to *β*‐nootkatol remains as irreversible because the values of *k*_2_ and *k*_−2_ are similar to each other as well as to that (*k*_6_) of by‐product. Based on the above discussion, the overall reaction scheme is simplified as shown in Fig. 7, and the corresponding rate equations are updated in Table 5.

**Table 5:** Simplified reaction rate equations

Reaction	Rate equation	
V→A	$\begin{array}{c} r_{1}=\frac{k_{1}K_{V}C_{V}}{1+K_{V}C_{V}+K_{A}C_{A}+K_{B}C_{B}+K_{N}C_{N}+K_{E}C_{E}+K_{\mathrm{By}}C_{\mathrm{By}}} \end{array}$	(17)
V↔B	$\begin{array}{c} r_{2}=\frac{k_{2}K_{V}C_{V}-k_{-2}K_{B}C_{B}}{1+K_{V}C_{V}+K_{A}C_{A}+K_{B}C_{B}+K_{N}C_{N}+K_{E}C_{E}+K_{\mathrm{By}}C_{\mathrm{By}}} \end{array}$	(18)
A→N	$\begin{array}{c} r_{3}=\frac{k_{3}K_{A}C_{A}}{1+K_{V}C_{V}+K_{A}C_{A}+K_{B}C_{B}+K_{N}C_{N}+K_{E}C_{E}+K_{\mathrm{By}}C_{\mathrm{By}}} \end{array}$	(19)
B→N	$\begin{array}{c} r_{4}=\frac{k_{4}K_{B}C_{B}}{1+K_{V}C_{V}+K_{A}C_{A}+K_{B}C_{B}+K_{N}C_{N}+K_{E}C_{E}+K_{\mathrm{By}}C_{\mathrm{By}}} \end{array}$	(20)
N→E	$\begin{array}{c} r_{5}=\frac{k_{5}K_{N}C_{N}}{1+K_{V}C_{V}+K_{A}C_{A}+K_{B}C_{B}+K_{N}C_{N}+K_{E}C_{E}+K_{\mathrm{By}}C_{\mathrm{By}}} \end{array}$	(21)
V→By	$\begin{array}{c} r_{6}=\frac{k_{6}K_{\mathrm{By}}C_{\mathrm{By}}}{1+K_{V}C_{V}+K_{A}C_{A}+K_{B}C_{B}+K_{N}C_{N}+K_{E}C_{E}+K_{\mathrm{By}}C_{\mathrm{By}}} \end{array}$	(22)

**Figure 8:**
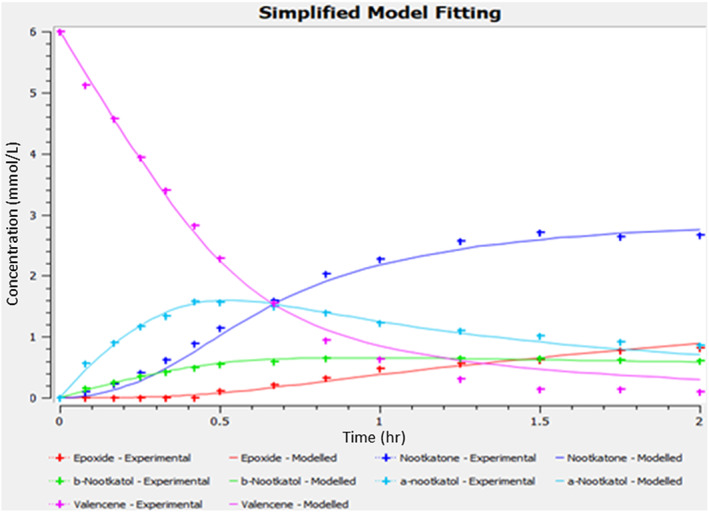
Fit between experimental data and model predictions using the simplified model (valencene concentration = 6 mmol L^−1^, enzyme concentration = 1.5 μmol L^−1^, aeration = 0 vvm, *f* = 2.5 Hz, *x*_o_ = 40 mm).

Figure 8 shows the fit between the experimental and model predictions using the simplified rate equations. The fitting is equally good with faster convergence. The simplified model is used thereafter.

From Table 4 we also note that the adsorption constants for *α*‐nootkatol, *β*‐nootkatol and nootkatone are similar and of minimal values when compared to those of valencene and epoxide, meaning that enzyme has a higher selectivity towards the intermediates and the main product, which is consistent with the kinetics.

### Effect of mass transfer

**Figure 9:**
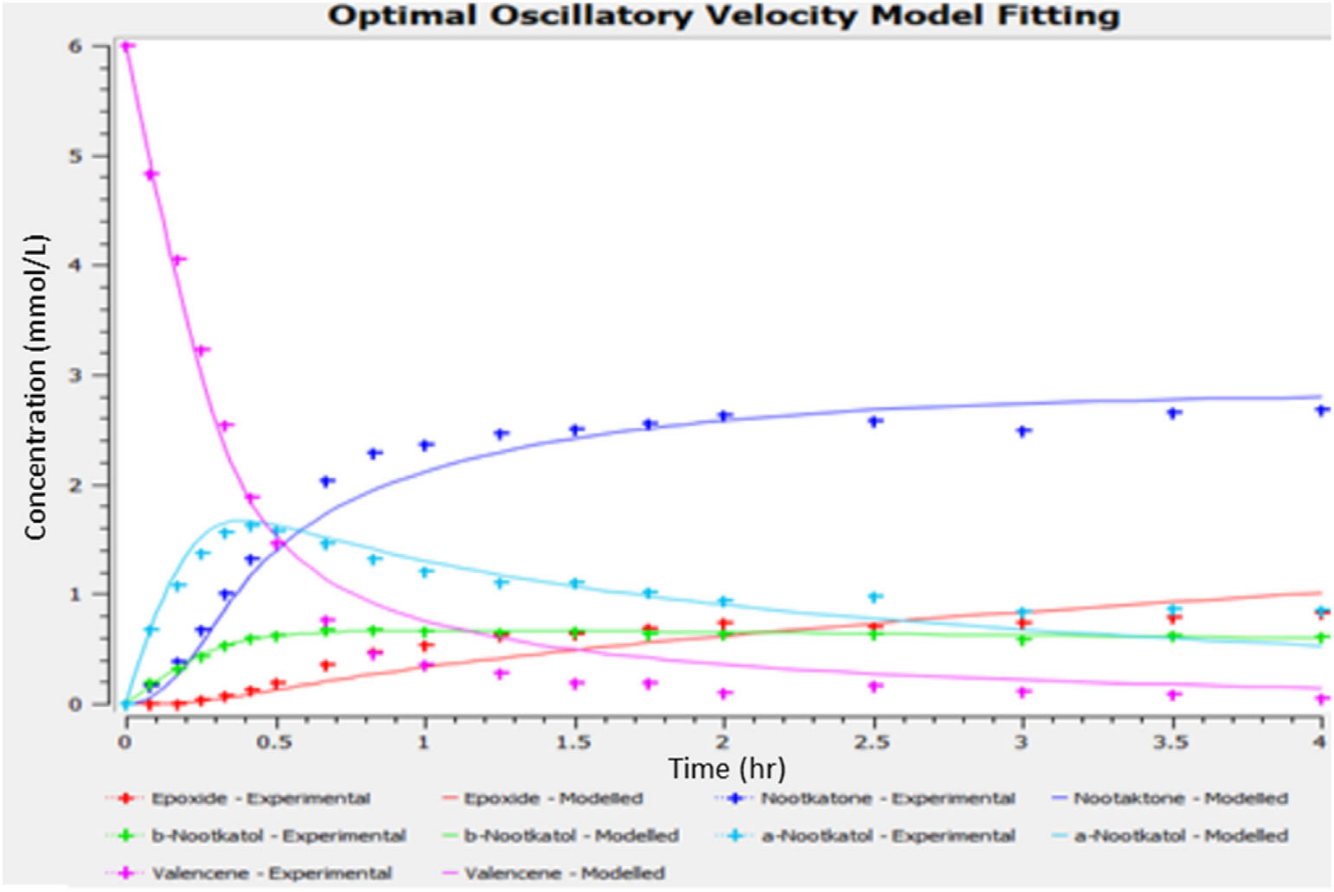
Fit between experimental data and model predictions for the optimal oscillatory velocity.

In the overall reaction scheme (Fig. 1), dissolved oxygen has a great influence on valencene transformation and can be enhanced by improved mixing.^26^ The mixing in the OBR is generated by the periodical generation and termination of eddies, controlled by the combination of oscillation amplitude (which controls the length of eddies) and the frequency (which controls the periodicity). Using the simplified kinetic model, the effect of oscillatory mixing, in terms of the oscillatory velocity (= *f* × *x*_o_ in mm s^−1^), on reaction kinetics is summarized in Table 6 and the fitting is shown in Fig. 9.

**Table 6:** Estimated kinetic parameters at different oscillatory velocities (valencene load = 6 mmol L^−1^, enzyme load = 1.5 μmol L^−1^, aeration rate = 0 vvm)

Velocity (mm s^−1^)	*k*_1_ (h^−1^)	*k*_2_ (h^−1^)	*k*_−2_ (h^−1^)	*k*_3_ (h^−1^)	*k*_4_ (h^−1^)	*k*_5_ (h^−1^)	*k*_6_ (h^−1^)
40.5	1.78	0.43	0.00	0.08	0.00	0.00	2.00
46.5	0.27	0.00	0.00	0.00	0.00	0.00	2.00
52.5	0.27	1.00	0.00	0.00	0.00	0.00	0.00
54	1.50	0.39	0.00	0.00	0.00	0.00	0.00
62	2.00	0.54	0.00	0.00	0.00	0.00	0.00
67.5	0.95	0.21	0.00	0.21	0.05	0.00	0.00
70	0.74	0.00	0.00	24.76	0.00	4.79	0.00
77.5	0.82	0.18	0.00	21.77	4.43	4.13	0.36
87.5	1.80	0.42	0.21	15.72	3.73	1.67	0.86
100	6.21	1.37	0.76	123.62	6.46	8.37	1.17
110	6.60	1.44	0.15	118.44	15.81	18.28	1.51
120	8.84	2.13	0.81	119.81	19.54	50.38	1.99
122.5	6.58	1.48	0.48	109.35	7.77	4.35	1.19
140	9.30	2.26	0.38	190.86	28.55	15.95	2.14
157.5	5.13	1.08	0.00	70.26	9.69	14.41	1.06

From Table 6, we can see that at low oscillatory velocities, the kinetic constants of the reactions of valencene to *α*‐nootkatol to nootkatone are generally low because the oscillatory motions were not strong enough to break up the bubbles and force interactions between molecules. At higher oscillatory velocities, increases in *k*_1_, *k*_2_, *k*_3_ and *k*_4_ are clearly seen with higher amounts of nootkatone being produced. These are the evidence that the steps are mass transfer controlled. The maximum production rate of nootkatone in a 200 mL working volume was 127.52 mg h^−1^ at 120 mm s^−1^ oscillatory velocity.

## CONCLUSIONS

We carried out an oxidation reaction to convert valencene to nootkatone using a proprietary enzyme (P450_BM3_) in an OBR, and obtained the concentration profiles of all species changing with time. A kinetic model based on the Langmuir–Hinshelwood kinetics method was employed to extract reaction kinetics. The good fits between the experimental data and the model predictions validate the model, confirming the reaction schemes and the orders of the reactions. The analysis of rate constants allows the multi‐reaction steps to be further simplified. We investigated the effect of mass transfer on kinetics: increasing the mixing intensity increases the rates of production of nootkatone via intermediates. This is consistent as the reaction is mass transfer controlled. The maximum production rate of nootkatone in a 200 mL working volume was 127.52 mg h^−1^ at an oscillatory velocity of 120 mm s^−1^.

## CONFLICT OF INTEREST

MH is an employee of Oxford Biotrans which has a commercial interest in the reaction described here. Both X‐WN and MC neither have a financial stake in nor received any payment from Oxford Biotrans for the work reported in this paper.

## Data Availability

Data are available on request from the corresponding author. The proprietary modified P450_BM3_ enzyme used in this work could be made available to other academic researchers upon request, subject to a materials transfer agreement with Oxford Biotrans.
